# Directional Summation in Non-direction Selective Retinal Ganglion Cells

**DOI:** 10.1371/journal.pcbi.1002969

**Published:** 2013-03-14

**Authors:** Syed Y. Abbas, Khaldoun C. Hamade, Ellen J. Yang, Scott Nawy, Robert G. Smith, Diana L. Pettit

**Affiliations:** 1 Dominick P. Purpura Department of Neuroscience, Albert Einstein College of Medicine, New York, New York, United States of America; 2 Department of Neuroscience, University of Pennsylvania, Philadelphia, Pennsylvania, United States of America; 3 Department of Ophthalmology and Visual Sciences, Albert Einstein College of Medicine, New York, New York, United States of America; Max Planck Institute of Neurobiology, Germany

## Abstract

Retinal ganglion cells receive inputs from multiple bipolar cells which must be integrated before a decision to fire is made. Theoretical studies have provided clues about how this integration is accomplished but have not directly determined the rules regulating summation of closely timed inputs along single or multiple dendrites. Here we have examined dendritic summation of multiple inputs along On ganglion cell dendrites in whole mount rat retina. We activated inputs at targeted locations by uncaging glutamate sequentially to generate apparent motion along On ganglion cell dendrites in whole mount retina. Summation was directional and dependent13 on input sequence. Input moving away from the soma (centrifugal) resulted in supralinear summation, while activation sequences moving toward the soma (centripetal) were linear. Enhanced summation for centrifugal activation was robust as it was also observed in cultured retinal ganglion cells. This directional summation was dependent on hyperpolarization activated cyclic nucleotide-gated (HCN) channels as blockade with ZD7288 eliminated directionality. A computational model confirms that activation of HCN channels can override a preference for centripetal summation expected from cell anatomy. This type of direction selectivity could play a role in coding movement similar to the axial selectivity seen in locust ganglion cells which detect looming stimuli. More generally, these results suggest that non-directional retinal ganglion cells can discriminate between input sequences independent of the retina network.

## Author Summary

Visual information is coded by the output of retinal ganglion cells. Through evolution retinal ganglion cells acquired unique properties that allowed them to transmit to the brain such signals as direction of movement. The quest for the cellular mechanism of the detection of movement by retinal ganglion cells has been the holy grail of research on direction selectivity. In this study we have found a mechanism that allows individual non-direction selective On retinal ganglion cells to code sequences of excitatory inputs moving either away or toward the soma. We observed that inputs moving away from the soma resulted in enhanced, supralinear EPSP summation. Evidence from computational modeling suggests that expression of a specific set of voltage-dependent channels in dendrites introduces nonlinearities that could give a ganglion cell the ability to code looming motion. We predict that in a retinal network, such a directional tuning mechanism, together with asymmetric presynaptic inhibition, could be the building block for the development of more complex detection of visual motion.

## Introduction

Regardless of their classification, virtually all ganglion cells receive input from multiple bipolar cells. For example, in the cat and guinea pig retina, as many as 150 bipolar cells synapse onto a single On type ganglion cells [1]–[3]. With the exception of the soma and very proximal dendrites, bipolar cells contact ganglion cells uniformly throughout the dendritic tree [1], [4]. Thus ganglion cells have the task of integrating synaptic input from multiple bipolar cells whose synapses are distributed throughout the entire dendritic tree before a decision to fire an action potential can be made [5], [6].

Much of our current knowledge of the dendritic integration of multiple excitatory inputs comes from modeling studies [7]–[10] as well as analysis of conductance changes evoked by illumination of ganglion cell receptive fields [11]. However, these studies do not address the rules regarding summation of closely timed inputs along single or multiple dendrites. Similarities in the local statistics of light in natural scenes [12] result in highly correlated activity of neighboring bipolar cells. Thus, as the eyes sample the visual world, individual ganglion cell dendrites are likely to be activated by cohorts of bipolar cells whose input with spatio-temporally correlated synaptic output. Theoretical work has suggested that there is a directional component to dendritic integration [13], and this has been confirmed in cortical neurons where activation sequence toward the soma (centripetal) produced more summation than the sequence directed away from the soma (centrifugal) [14]. However, it is unclear whether the same rules for dendritic integration apply to retinal ganglion cells.

To test for direction-dependent summation of excitatory postsynaptic potentials (EPSPs) in On ganglion cells, we activated multiple distinct loci along a dendrite with targeted local photolysis of caged glutamate. Surprisingly, we found that summation was supralinear for centrifugal input, while centripetal activation resulted in linear summation. Hyperpolarization activated cyclic nucleotide-gated (HCN) channels have been shown to modulate summation and firing in a number of brain regions making them excellent candidates for modulators of dendritic integration in ganglion cells [15]–[20]. We found that blockade of HCN channels increased overall summation, and eliminated the directional component of summation. Further, the effect of blockade was most pronounced in the distal dendrites suggesting that the density of HCN current increases with distance from the soma. Our results show that intrinsic properties of the ganglion cell allow non-direction selective cells to code specific input sequences. This directional summation of EPSP is similar to that seen in starburst amacrine cells where centrifugal stimuli produce larger Ca^2+^ responses [21], [22]. Our results suggest that this fundamental mechanism for directional summation is an essential building block across multiple cell types and species for generating a traditionally direction selective cell.

## Methods

### Ethics statement

Sprague Dawley rats were purchased from Charles River Laboratories (Wilmington, PA, U.S.A.) and housed in the Albert Einstein College of Medicine Animal Care Facility. Rats were subjected to 12 h light/12 h dark cycles, and were fed a standard chow diet. All procedures were performed according to a protocol approved by the Institute for Animal Studies of the Albert Einstein College of Medicine.

### Physiology

Whole mounts of rat retina (P14–18 and P21–35), were prepared from dark adapted (1 hr) animals. Animals were anesthetized with isoflurane applied to gauze attached to the top of a closed chamber. This results in loss of consciousness within a minute. Animals were then decapitated, and subsequent surgery and cell recordings were performed in dim red light. The eyes were enucleated and retinal dissections were performed in oxygenated physiological saline (in mM: 119 NaCl, 2.5 KCl, 1.3 MgCl_2_, 2.5 CaCl_2_, 1 NaH_2_PO_4_, 26.2 NaHCO_3_, 11 glucose). The eyecup was cut into two pieces, the retina removed with forceps, and transferred to a recording chamber.

Whole-cell recordings were made from ganglion cells using a patch pipette filled with (in mM): 135 KMeSO_3_, 5 KCl, 1 CaCl_2_, 5 EGTA, 10 glucose, 2 ATP, 0.3 GTP, 10 HEPES (pH to 7.2) and 100 µM Oregon-green BAPTA. The retina was superfused at 30°C with oxygenated physiological saline. Recordings were performed under infrared conditions using a CCD video camera fitted with IR filter and macro lenses. A halogen source was used for light stimuli. Cells were selected for patching based on large soma size. This approach yielded ∼85% On cells. At the beginning of each experiment ganglion cell subtype was identified using a light stimulus in the dark adapted retina. Subsequent use of the 405 nm uncaging or 488 imaging laser changed adaptation state. The use of caged compound allowed us to isolate the postsynaptic response independent of presynaptic circuitry. Only one cell was obtained from a preparation insuring all cells were initially dark adapted.

Light evoked EPSP responses were kept subthreshold by placing 3 or 4 log unit neutral density filters in the light path, and varying stimulus duration (range 10–200 ms). Individual ganglion cells were then imaged with an Olympus Fluoview 1000 confocal microscope using a 40× water-immersion objective [23]. Laser light was limited to brief exposures sufficient to identify the dendritic target for the uncaging beam. If holding current exceeded 100 pA the experiment was terminated. A Z-series of confocal images were collected at the completion of the experiment. 200–500 µM MNI-caged-glutamate (Tocris, Ellisville, MO) was added to the external solution. (2R)-amino-5-phosphonovaleric acid (APV; 50 µM; Tocris, Ellisville, MO), and ZD7288 (100 µM; Tocris, Ellisville, MO) were used to block NMDA and HCN channels, respectively. Recordings were accepted only if the holding current was less than −150 pA when ganglion cells were voltage clamped at −67 mV. Electrophysiology data were collected and analyzed off line with pClamp (Molecular Devices, Sunnyvale, CA). The “HCN isolation solution” contained (in mM): TTX 0.1, BaCl_2_ 1, NiCl_2_ 0.1, D-AP5 0.05, NBQX 0.01, picrotoxin 0.05, TPMPA 0.05, strychnine 0.001; NaH_2_PO_4_ was omitted when Ba^2+^ and Ni^2+^ were present to avoid precipitation. TTX, BaCl_2_, NiCl_2_, APV, NBQX, picrotoxin, TPMPA, and strychnine were used to block sodium, inward rectifying potassium, calcium, *N*-methyl-d-aspartate (NMDA), AMPA receptor, GABA_A_, GABA_C_ and glycine receptors, respectively [24], [25].

### Uncaging

An Olympus Fluoview 1000 confocal microscope equipped with a 405 diode laser (1–3 ms pulses) was used for uncaging MNI-caged glutamate (200–500 µM) at multiple targeted regions of the dendrite (0.5 ms minimum delay between locations). Laser power and uncaging duration were kept constant for the duration of an experiment. For all experiments we obtained 8 trials for each uncaging sequence.

### Retinal ganglion cell cultures

Retinas were isolated from newborn (P0) rats after cryoanesthesia and were incubated for 45 min at 37°C in DMEM with HEPES (Mediatech, Washington, DC), supplemented with 6 units/ml papain (Worthington, Freehold, NJ) and 0.2 mg/ml cysteine. Papain was then inactivated by replacing the enzyme solution with complete medium composed of DMEM, 5 mM HEPES, 0.1% Mito^+^ serum extender (Collaborative Research, Bedford, MA), 5% heat-inactivated fetal calf serum (HIFCS), 0.75% penicillin-streptomycin-glutamine mix (Life Technologies). Osmolarity was adjusted to 300 mOsm by addition of distilled water. Retinas were triturated through a fire-polished Pasteur pipette, plated onto glass coverslips pretreated with poly-D-lysine (0.1 mg/ml), and maintained in complete medium supplemented with 15 mM KCl. 72 hr after plating, cells were treated with the antimitotics 5-fluoro-2-deoxyuridine (0.01 mg/ml) and uridine (0.026 mg/ml) for 24 hrs. Cells were used for recording or immunohistochemistry at 11–14 days *in vitro*.

### Immunohistochemistry

Cultured retinal ganglion cells were fixed for 10 min in 4% paraformaldehyde (PFA), rinsed with Tris-buffered saline (TBS), and blocked for one hour with TBS containing 4% bovine serum albumin (BSA), and 0.1% Triton-X 100. After blocking, cells were incubated with primary antibodies (chicken anti-MAP2, 1∶1000, Millipore, Billerica, MA; rabbit anti-GluR2, 1∶200, Millipore; anti-HCN1, anti-HCN2, and anti HCN4 raised in mouse, 1∶200, NeuroMab, Davis, CA) for 1 hour. Cells were washed in TBS and incubated with fluorescently-conjugated secondary antibodies (Alexa Fluor 488 goat anti-chicken IgG, 1∶1000, Life Technologies, Grand Island, NY; Cy-3 conjugated donkey anti-mouse IgG (H+L), 1∶500; Cy-5 conjugated donkey anti-rabbit IgG (H+L), 1∶500, Jackson ImmunoResearch Laboratories, West Grove, PA). After washing, coverslips were mounted for imaging. Retinal ganglion cells were imaged using a Hamamatsu Orca ER camera mounted on an inverted Nikon fluorescent microscope with a 60× Plan Apo lens. The strength of the excitation light and length of exposure time were kept constant for precise comparisons. Images were background-subtracted, and analyzed using Metamorph software (Molecular Devices, Sunnyvale, CA). No labeling was observed in control slices in which the primary antibody was omitted. HCN1 immunoreactivity was quantified by comparing the average intensity value for HCN1 labeling in proximal (0–50 µm) and distal (>100 µm) dendrites.

### Computational models

Morphologies for ganglion cells were derived from live images of ganglion cells taken after recordings. An image of the dendritic arbor was digitized by tracing each dendrite manually or by a semi-automatic algorithm, similar to previous models [10], [26]. The dendritic arbor was divided into regions (proximal, middle, and distal) that could be assigned different biophysical properties. For some simulations, the cell was given ion channels defined by Markov sequential-state models, with various combinations of densities of 3 channel types (K_HCN_, Na, K_dr_). Channel densities and other biophysical properties (Rm, Ri) were specified for each region of the cell. The standard set of biophysical properties is listed in Table 1. For some models, we set the channel densities uniform across the cell, but for others, we specified a gradient of channel densities as a function of distance from the soma. In addition, an offset voltage and kinetic rate multiplier could be specified for the rate functions for each channel type.

**Table 1:** Values of the standard set of biophysical parameters for regions of the ganglion cell.

Parameter	Distal dendrite	Dendrite	Proximal Dendrite	Soma
**Na _v_ 1.2**	20	20	20	5
**K_dr_**	5	5	5	2
**K_hcn_**	0.005	0.001	0.0005	0
**V_rev_**	−0.068	−0.068	−0.068	−0.068
**R_m_**	28000	28000	28000	28000

The table shows the parameters for the GC model. In the leftmost column Na_v_ 1.2, K_dr_, K_hcn_ are channel densities given in mS/cm^2^. V_rev_ is the membrane leak reversal potential (volts). R_m_ is membrane (leak) resistance in Ωcm^2^. For all morphologies R_i_ = 200 Ωcm.

The model of the HCN channel was taken from the literature [27], [28]. Further modifications were performed to match results obtained from voltage step responses and tail current experiments. The rate of activation and deactivation for HCN were decreased by a factor of 0.33 and 2 respectively, to approximately match the data in whole cell recording. We simulated the uncaging of glutamate with a synaptic model connected to a presynaptic compartment that was voltage-clamped. The voltage clamp was directly set by the intensity of a light stimulus at the local (X, Y) location. The background light “intensity” was set to the resting potential for the pre-synaptic compartment, and the stimulus “contrast” generated a depolarizing pulse of several mV. This voltage controlled an exponential release function with 2 mV per e-fold change. To approximate the time course of the real responses, the released neurotransmitter was passed through a first-order temporal filter with separate time constants for rise and fall, set to 75 ms and 125 ms respectively. The filtered glutamate release bound to post-synaptic AMPA receptors modeled using a markov diagram, derived from [29]. The reversal potential for the AMPA conductance was set to 0 mV. Thus, synaptic conductance was modulated by the “contrast” of the stimulus to the presynaptic compartment, to vary the amplitude of EPSPs recorded in the simulated ganglion cell. Synapses were positioned at regular intervals (∼10 µm, with regularity (m/SD) = 8) over the dendritic tree by an automatic algorithm. Stimulus pulses were given to specific points on a dendrite for a specified duration (3 ms) and inter-pulse interval (20 ms). Sets of 3 to 5 locations were selected along the major dendrites at different radial distances from the soma. The simulations were performed using the Neuron-C simulation language [26], [30].

For most model runs, we saved the model's state parameters (voltages, channel occupancies, etc) after initial equilibrium, just before the first stimulus (the sequence of “uncaging” in centripetal or centrifugal directions), and restored the model parameters after the first stimulus and just before the second. This obviated any interaction between the timing of the first stimulus and the second. When we removed the “restore” of the model's state before the second stimulus, we found that within an interval of 500 ms there was a noticeable effect of the first stimulus upon the second, consistent with the slow activation/deactivation of HCN. Because the data from the real cells was obtained with long intervals (more than 1s) between directions, we utilized the “save”, “restore” feature of the simulator for all other model runs. Directional summation (DS) was calculated for amplitude as (Vpeak away – Vpeak toward)/(Vpeak toward – Vrest) * 100, and (Charge away – Charge toward)/Charge toward * 100 for charge.

## Results

### Photolytic responses

**Figure 1:**
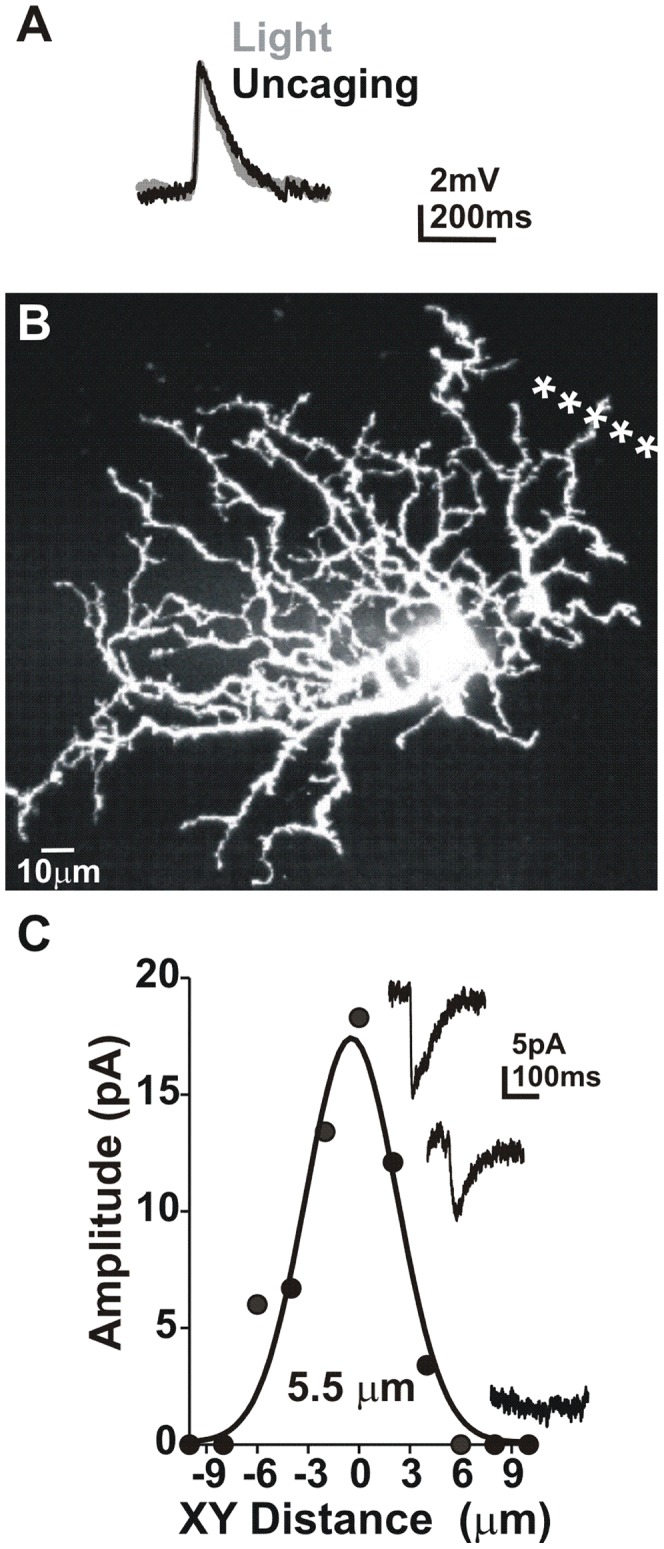
Uncaging EPSP properties. **A**) Scaled light and uncaging EPSPs (traces are averages of 5 trials). **B**) Confocal image of ganglion cell dendrites. **C**) A plot of XY resolution for the cell in B. * = photolysis.

We used local photolysis of caged glutamate to examine location and timing-dependent differences in summation of dendritic input. Whole-cell voltage and current clamp recordings were obtained from On ganglion cells in whole mounts of dark-adapted rat retina. A Fluoview 1000 equipped with a 405 diode laser was used to uncage glutamate over ganglion cell dendrites in whole mount retina (see Methods). Postsynaptic response size was regulated by adjusting laser intensity to insure that the responses were subthreshold (1–4 mV) and did not initiate action potentials. This approach elicited EPSPs with a time course that was similar to the light responses (Figure 1A). Measured decay times were 98.0±14.9 ms (light; N = 10 On cells) and 109.0±14.4 ms (uncaging; N = 9 On cells) and the 10–90% rise time was 40.3±8.2 ms (light; N = 10 On cells) and 48.6±5.9 (uncaging; N = 9 On cells). The differences in decay and rise time were not significant (t-test; p>0.05). This suggests that while the uncaging stimulus may not be identical to light stimuli, the EPSPs are in a physiologically relevant range.

The size of the uncaging area was measured by uncaging at 2 µm intervals perpendicular to the dendrite (Figure 1B). Current amplitude at each location was plotted against distance from the dendrite and fit with a Gaussian function. The average half-maximal width of that fit was then used as a measure of lateral (XY) resolution. On average, the half-maximal width of the Gaussian fit was 5.7±0.5 µm (N = 7 cells; Figure 1C). As a result, individual uncaging locations were separated by a minimum of 20 µm, insuring no overlap between sites.

### Dendritic summation is supralinear in On retinal ganglion cells

**Figure 2:**
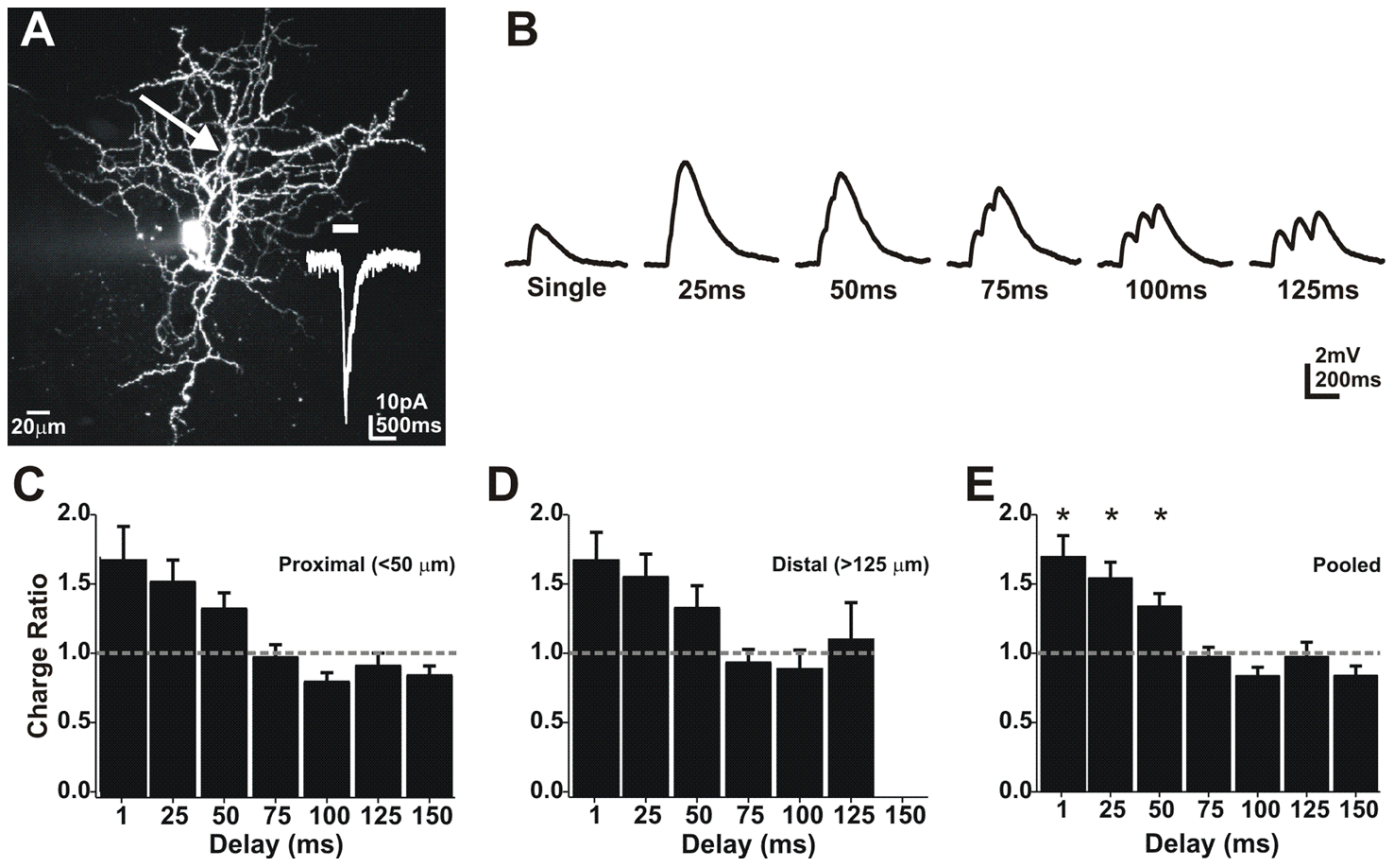
Dendritic summation is supralinear in retinal ganglion cells. **A**) A confocal image of an On ganglion cell filled with Oregon green-BAPTA (100 µM). Trains of 3 uncagings were delivered to an individual location (arrow) at varying intervals (1–125 ms). Lower left trace is the light response, bar indicates light duration. **B**) Summed depolarization uncaging responses (traces are averages of 8 trials). **C**) A plot of the total charge for summed depolarizations/sum of 3 individual depolarization charges for locations <50 µm from the soma. **D**) Locations >125 µm from the soma. **E**) Pooled data (ANOVA p<0.001; N = 11 On cells).

To determine the timing rules underlying summation in On ganglion cells we measured the linearity of summation at an individual dendritic location in whole mount retina. To evaluate the timing rules governing summation, trains of three uncaging pulses were delivered at varied delays (1–125 ms), to the same location (single pixel) (Figure 2A, B). Delays were presented in random order to avoid use-dependent artifacts. The amount of summation for each delay was then calculated by dividing the area (charge) or amplitude of the summed EPSP response by 3 times the charge or amplitude for a single EPSP response. As a result, a summed response with a total charge equal to 3X that of a single response would have a summation ratio of 1, indicating linear summation. Ratios larger or smaller than 1 indicated supra or sub linear summation, respectively.

Previous work in cortical neurons has suggested that summation is not uniform over the length of the dendrite [31]. To look for local differences in ganglion cell summation we compared summation at proximal locations (<50 µm) to summation from distal dendritic locations (>50 µm; range 80–250 µm; Figure 2C, D). Surprisingly, we found no significant differences between the two groups (paired t-test; p>0.05; N = 9 On cells), suggesting summation in ganglion cells is uniform within 250 µm along the length of the dendrites. Delays of 50 ms or less produced supralinear responses while longer delays resulted in near linear summation (Figure 2E; N = 11 On cells). Summation ratios for short delays (1–50 ms) were not significantly different from each other (ANOVA; p>0.05; N = 11 On cells). However, delays of ≤50 ms resulted in significantly more summation than longer delay times (>50 ms; ANOVA; p<0.0025). Therefore, we used 50 ms delays for all subsequent summation experiments.

### Summation is direction-dependent

**Figure 3:**
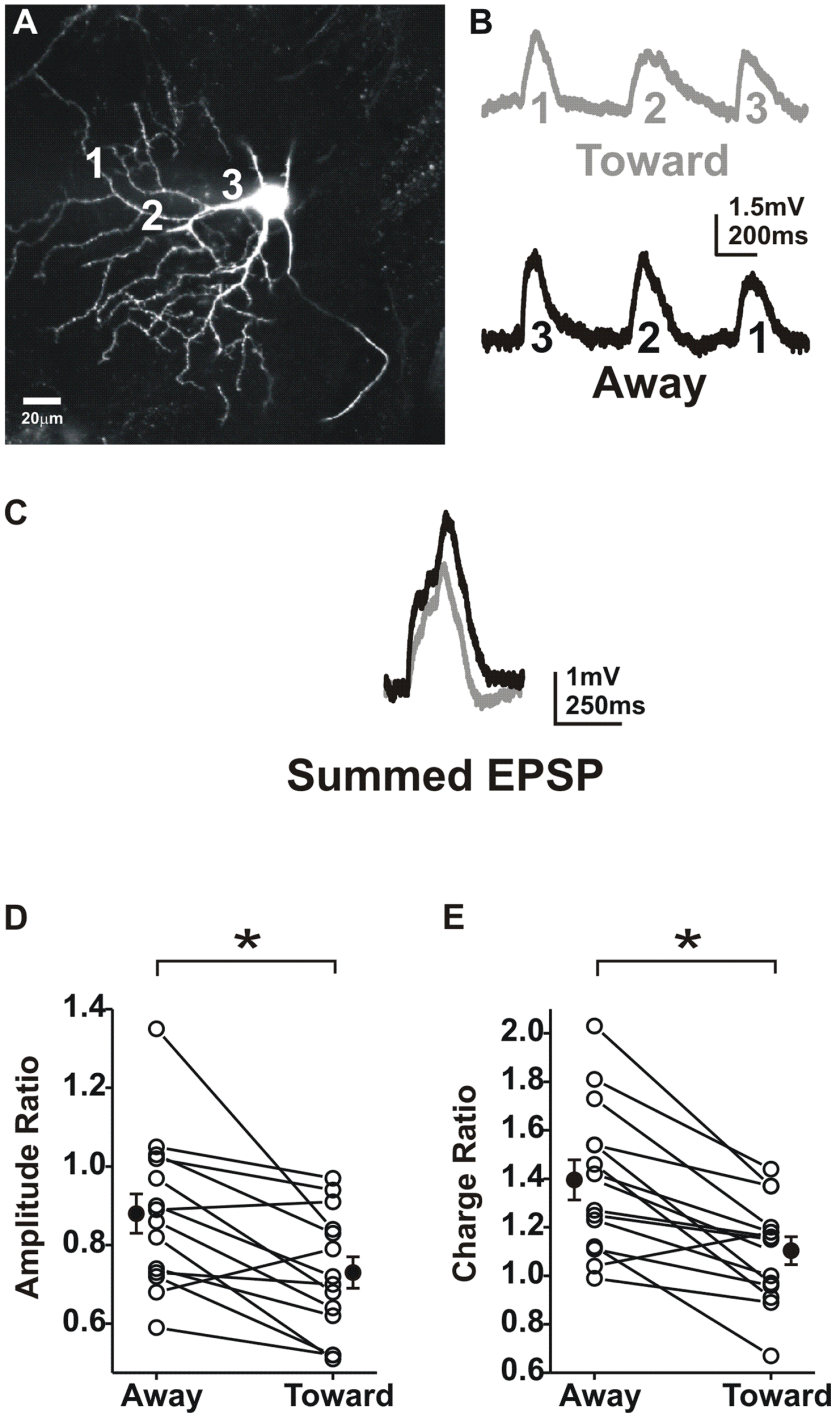
Dendritic summation has a directional component. **A**) A single confocal plane of an On cell showing uncaging locations over 3 dendrites. **B**) Individual EPSPs moving either away (black) or toward (grey) the soma for the dendrite in A (400 ms delay between locations; averages of 8 trials). **C**) Summed EPSPs moving away (black) or toward the soma (50 ms delay between locations; averages of 8 trials). **D**) A plot of the amplitude ratio (summed EPSP/3x single EPSP). 0.88±0.05 away, 0.73±0.04 toward; paired t-test p<0.005; N = 14 On cells; P14–18). **E**) A plot of the charge ratio for summed depolarizations moving away from or toward the soma. 1.39±0.08 away, 1.10±0.05 toward; paired t-test; p<0.005; N = 15 On cells.

Theoretical work has suggested that summation would be greater for inputs moving toward the soma than for those moving away from the soma [13]. In support of this hypothesis a recent study of cortical neurons demonstrated that summed EPSPs were larger in amplitude for input sequence directed toward the soma (centripetal) [14]. To test for a directional component to summation in ganglion cells, we activated multiple regions of the dendrite in both directions (Figure 3A).

We expected that somatic responses elicited from distal dendrites would be smaller and slower than those from proximal dendrites (as measured at the soma). This is due to membrane leak, axial resistance and membrane capacitance, which increases with dendritic length. Surprisingly, we found the glutamate responses were relatively uniform along and between dendrites (Figure 3B). Across all cells, depolarization amplitudes showed no significant location-dependent differences (ANOVA; p>0.05; N = 15 cells). As ganglion cell dendrites are heavily branched it was possible that multiple dendrites could be activated by the uncaging beam, given that measured axial (Z) resolution was 40±4.7 µm (N = 5 cells). While this could have been a potential interpretation problem, the uniform response amplitude argues against this possibility as activation of multiple dendrites would produce random and significant irregularity in EPSP magnitude. These results also suggest that ganglion cell dendrites employ mechanisms that compensate for electronic decay to insure input strength is accurately represented at the soma. This is consistent with predictions based on computational modeling which suggest that for a physiologic range of membrane resistance and moderate strength stimuli, inputs are equally effective at all points along the ganglion cell dendritic tree [9].

We found that ganglion cell summation recorded at the soma was directional, but unlike cortical neurons, it was larger for input sequences directed away from soma (centrifugal; Figure 3C). To measure the linearity of summed EPSPs, individual EPSP amplitudes and the integrated charge of each waveform were arithmetically summed and amplitude and charge ratios were calculated for summed EPSPs (summed EPSP/3x single EPSP; plots of raw amplitude and charge can be found in Figure S1). Response amplitude ratios were significantly larger for inputs moving away from the soma (Figure 3D). This was also true for the charge, which had a significantly greater supralinear ratio for away directed inputs (Figure 3E).

**Figure 4:**
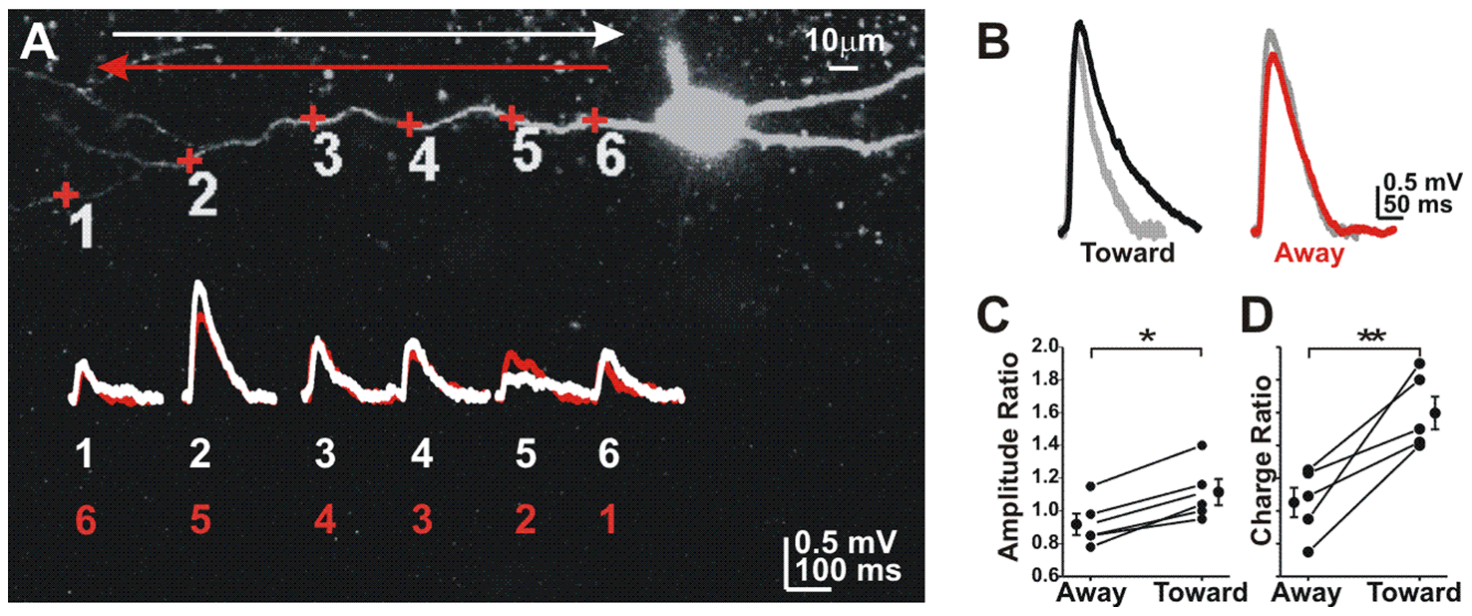
Cortical neurons favor centripetal summation. **A**) A hippocampal oriens lacunosum moleculare cell with uncaging locations (+) and EPSPs (average of 5 trials) for each location moving toward or away from the soma. **B**) Expected (calculated) charge for multiple activation (grey) vs. actual summed EPSP. **C**) Summation ratio for peak amplitude. **D**) Summation ratio for total charge. (* paired t-test; p<0.05 N = 6 cells; ** p<0.025; N = 5 cells).

Summation experiments were repeated using older animals (P22–35) to examine summation in mature retina. Again, the EPSP amplitude ratio was larger for sequences moving away from the soma (0.69±0.06 away, 0.6±0.06 toward; paired t-test p<0.01; N = 11 cells). The same was true for the charge ratio (1.32±0.37 away, 1.16±0.36 toward; paired t-test p<0.01; N = 9 cells). When summation for multiple dendrites on the same cell was examined we found similar ratios for all dendrites (N = 6 cells).In contrast, we found that summation in hippocampus oriens lacunosum moleculare interneurons was larger when moving toward the soma (Figure 4.), similar to what was reported in cortical neurons [14]. These results suggest that retinal ganglion cells have unique integration properties setting them apart from cortical neurons, consistent with their specialized function. This radial directionality is similar to that exhibited by starburst amacrine cells where centrifugal stimulation produces increased dendritic calcium signals [21], [22].

The directional summation (DS) we observe could be a network property rather than an intrinsic property of the ganglion cell. For example, neighboring amacrine cells could be activated by the free glutamate, changing the inhibitory input to ganglion cells. To test this possibility we repeated the experiment in Figure 3 by blocking inhibition. The addition of Picrotoxin (100 µM) and Strychnine (1 µM) to block GABA-A and Glycine receptors, respectively, resulted in unstable spontaneous activity, which rendered the data uninterpretable. Thus, we only included Picrotoxin in the bath solution to block inhibition, and found that peak amplitude and charge increased in both directions (28.2±5% (away) and 19.6±3% (toward)). However, picrotoxin had no effect on DS with an amplitude ratio of 1.27±0.08 (away) vs. 1.10±0.05 (toward) and charge ratios of 1.2±0.05 (away) vs. 1.12±0.04 (toward; t-test; p<0.05; N = 7 cells), suggesting inhibitory inputs alone are not responsible for DS.

### Directional summation in culture

**Figure 5:**
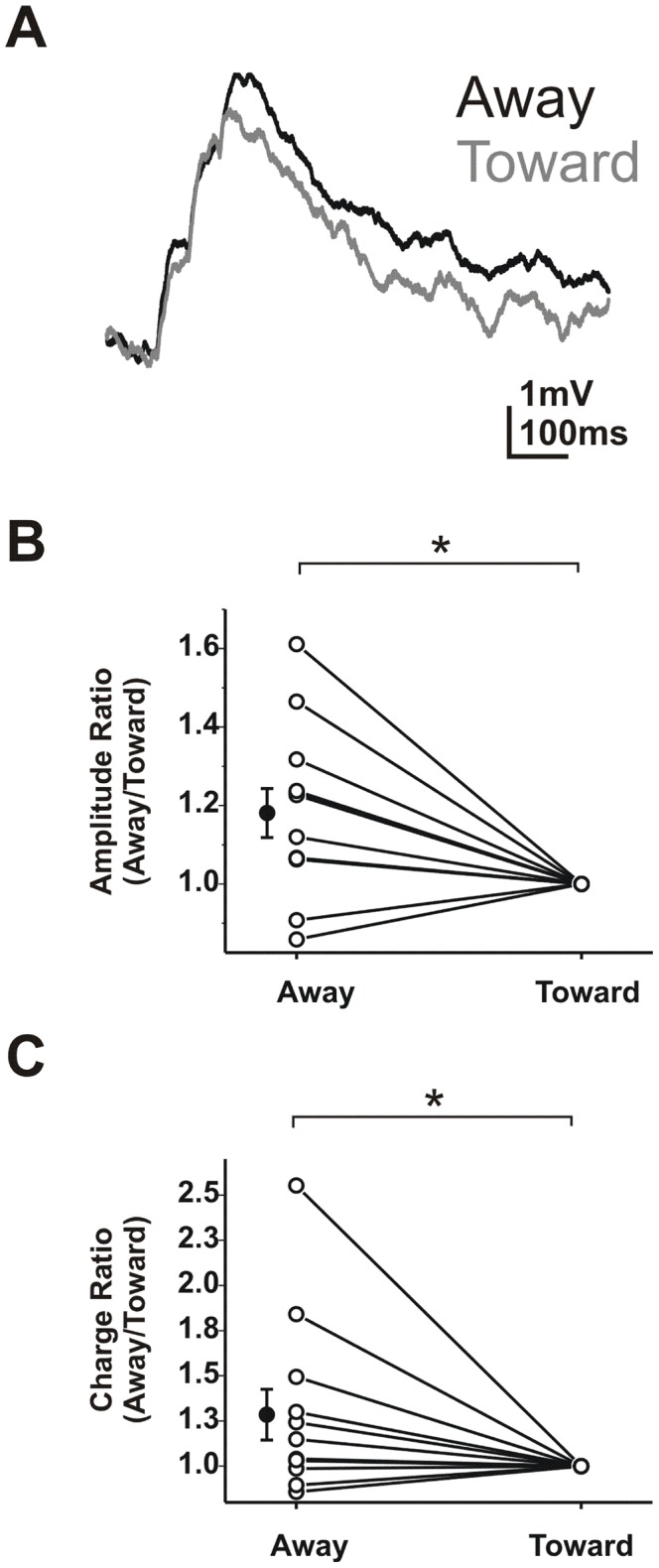
Direction selectivity is maintained in culture. **A**) Summed EPSPs from cultured ganglion cells. **B**) Normalized ratio of the peak EPSP amplitude for both directions. **C**) Normalized ratio of charge ratio in both directions.

As an additional test of network participation in DS we looked at summation in primary retina cultures. Whole-cell current clamp recordings were obtained from ganglion cells 8–21 days in culture and summation was measured in both directions. Figure 5A shows representative summed EPSPs confirming that DS was reconstituted in the cultures. Amplitude and charge ratios for culture experiments were calculated as away/toward. This was necessary because cultured neurons were difficult to hold for the lengthy time needed to acquire single and summed responses. For peak amplitude, summed EPSPs were larger moving away from the soma (1.18±0.06 vs. 1; paired t-test; p<0.05; N = 10 cells; Figure 5B). Charge was also higher moving away from the soma (1.29±0.14 vs. 1; paired t-test; p<0.05; N = 11 cells; Figure 5C). These results suggest that sequence-dependent summation is an intrinsic property of the ganglion cell rather than the network.

### Centrifugal activation increases excitability

**Figure 6:**
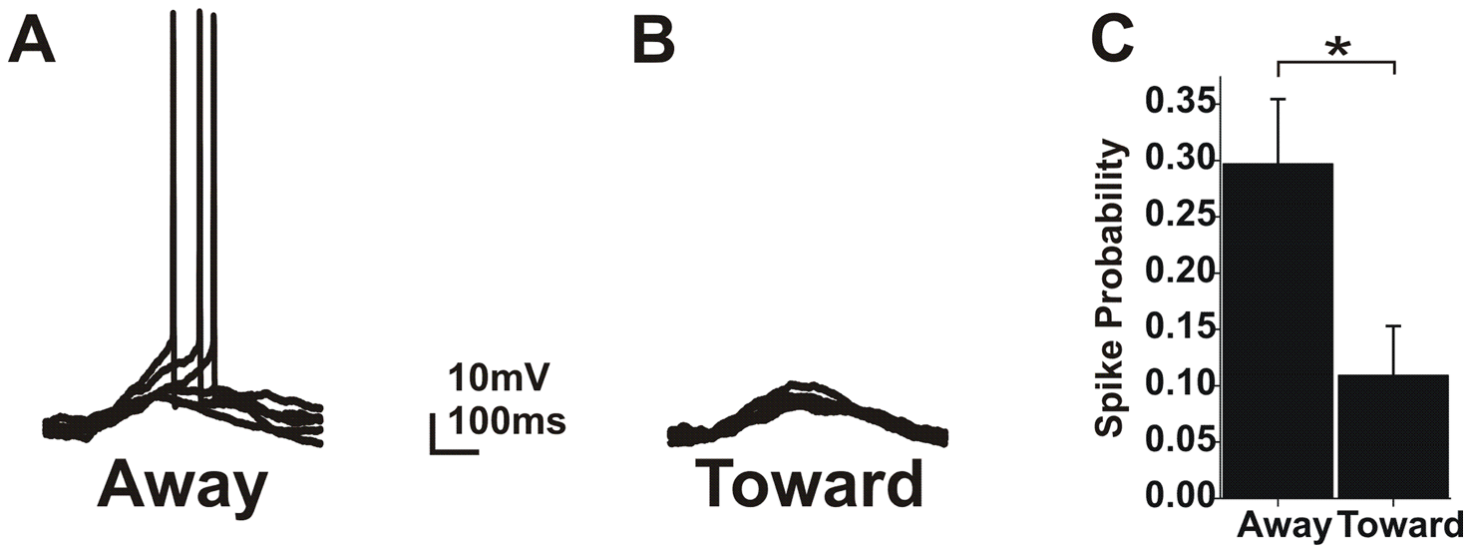
Directional summation increases excitability for centrifugal sequences. **A**) 5 consecutive traces showing suprathreshold responses for the away activation sequence. **B**) Matched traces for activation moving toward the soma. **C**) A plot of the average spike probability in both directions for all experiments showing suprathreshold activity.

Summation moving away from the soma was 26% larger than summation moving toward the soma in whole-mount retina. While this is a significant difference, the physiological importance of this increase is unclear. To determine whether this increase in summation translates to an increase in excitability we examined spike probability as a function of activation sequence. While we chose uncaging parameters intended to elicit subthreshold glutamate responses, a subset of experiments exhibited suprathreshold responses. For all cells exhibiting suprathreshold activity, we counted the number of suprathreshold trials for centripetal and centrifugal activation and calculated spike probability. Overall, spike probability was significantly higher when moving away from the soma (paired t-test; p<0.05; N = 9 cells; Figure 6A, B). Spike probability for activation moving away from the soma was 0.28±0.05 and 0.1±0.04 when moving toward the soma (Figure 6C). These results suggest that input sequence can have a significant impact on cell excitability and function for near threshold conditions.

### Mechanisms underlying directional summation

**Figure 7:**
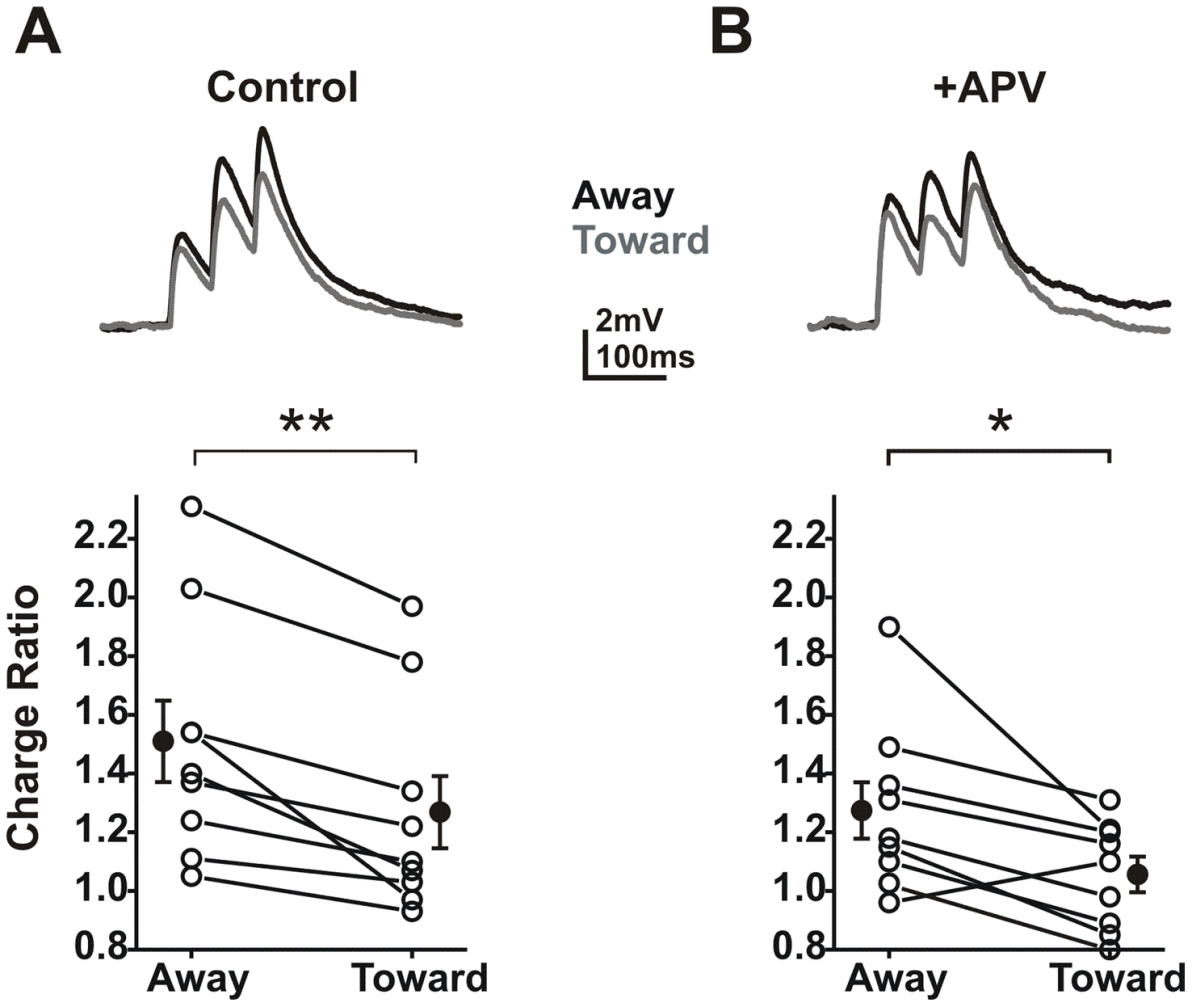
NMDA receptors increase summation but do not confer directionality. **A**) Summed traces (average of 8 trials) for activation of 3 locations along a single dendrite under control conditions. Lower: A plot of the charge ratio for all experiments. Summation was larger in the away direction (** paired t-test; p<0.01; N = 9 cells). **B**) Traces from the same 3 locations after application of APV (50 µm). Lower: A plot of the charge ratio for all experiments. Summation was still larger in the away direction (* paired t-test; p<0.05; N = 9 cells).

While the mechanisms responsible for creating DS are unclear, a likely possibility is the non-uniform expression of ion channels along dendrites. For example, the NMDA receptor has slow decay kinetics which can lengthen the integration time window and increase summation. If these channels were not uniformly distributed, location and direction-dependence of summation could result. In fact, when NMDA receptors were blocked with APV, EPSP summation was reduced and directionality eliminated in cortical neurons [14]. To determine the role of NMDA receptors in ganglion cell summation we compared summation before and after blockade of NMDA receptors with the antagonist APV (50 µM) in whole mount retina. APV reduced mean charge and amplitude for individual EPSPs, however, this difference was not significant (paired t-test; p>0.05; N = 9 cells). APV also reduced the summation ratio for charge from 1.51 (away) and 1.27 (toward) to 1.27 (away) and 1.05 (toward). While modest (15.6 and 17.3%), this reduction was significant (Figure 7A, B; paired t-test; p<0.025; N = 9 cells). Although APV reduced overall summation, it did not eliminate the directional component as summation remained significantly larger for activation sequences moving away from the soma (Figure 7). These results suggest that while NMDA receptors can enhance summation, they do not play a substantial role in establishing the direction-dependence of summation. We observed no difference in the effect of APV on proximal (<50 µm) vs. distal (>50 µm; range 80–250 µm) regions of dendrite, suggesting that NMDA receptor density was uniform.

### HCN channels modulate distal excitatory inputs

Hyperpolarization activated cyclic nucleotide-gated (HCN) channels are partially open at typical resting potentials where they depolarize the cell, decrease input resistance, and shorten the decay of synaptic signals, effectively reducing summation. Previous work has shown that these channels modulate EPSP summation and initiation of action potentials in a broad range of brain structures including hippocampus, brain stem, inferior colliculus and cortex [15]–[20]. In the hippocampus HCN current density is not uniformly distributed, increasing in density with distance from the soma [15]. Although mammalian retinal ganglion cells are thought to express these channels [26], [32], their distribution pattern has yet to be determined. If channel density is location-dependent, it could influence the observed directionality of summation in ganglion cells. To determine HCN channel density and their role in summation, we measured EPSP charge from whole mount retinal ganglion cells in the presence and absence of the HCN channel blocker ZD7288 (100 µM).

**Figure 8:**
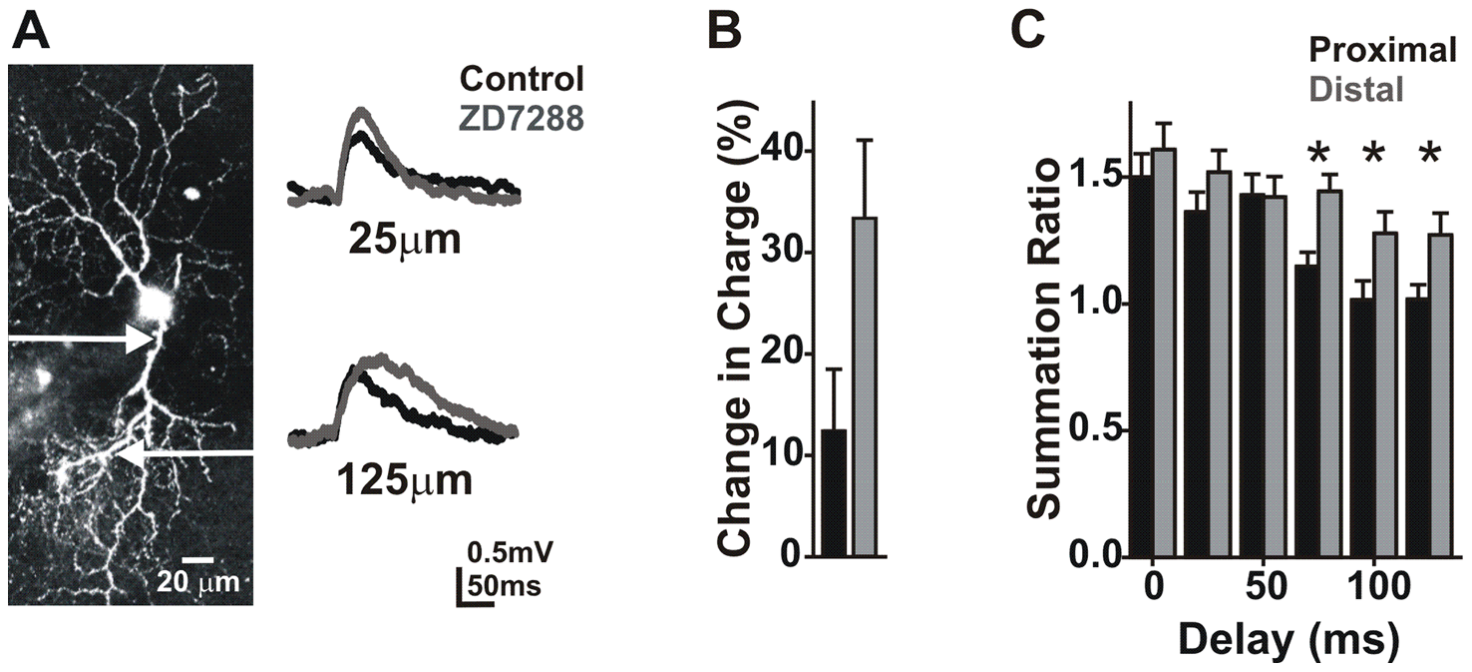
Blockade of HCN channels increases distal EPSP size. **A**) Traces (average of 8 trials) for proximal and distal locations before and after application of ZD7288 (100 µM) to block HCN channels. **B**) A plot of the increase in charge in both directions after ZD7288 application. **C**) Summation ratio for three uncaging pulses to the locations in A, with ZD7288 in the bath (*ANOVA; p<.05; N = 8 cells).

Blockade of HCN channels increased single trial EPSP charge and amplitude (Figure 8A, B). We hypothesized that under control conditions HCN should reduce summation by decreasing input resistance and decay time, reducing the integration time window. Regions of the dendritic arbor with higher HCN conductance should be more strongly affected than regions with reduced HCN conductance. Consistent with this possibility we found the effect of HCN blockade was largest in the distal dendrites (>50 µm; range 75–275 µm; N = 8 cells). Following HCN blockade with ZD7288, charge increased by 33.3±7.7% vs. 12.5±6.0% at proximal sites (Figure 8B; <50 µm; paired t-test; p<0.025; N = 8 cells).

**Figure 9:**
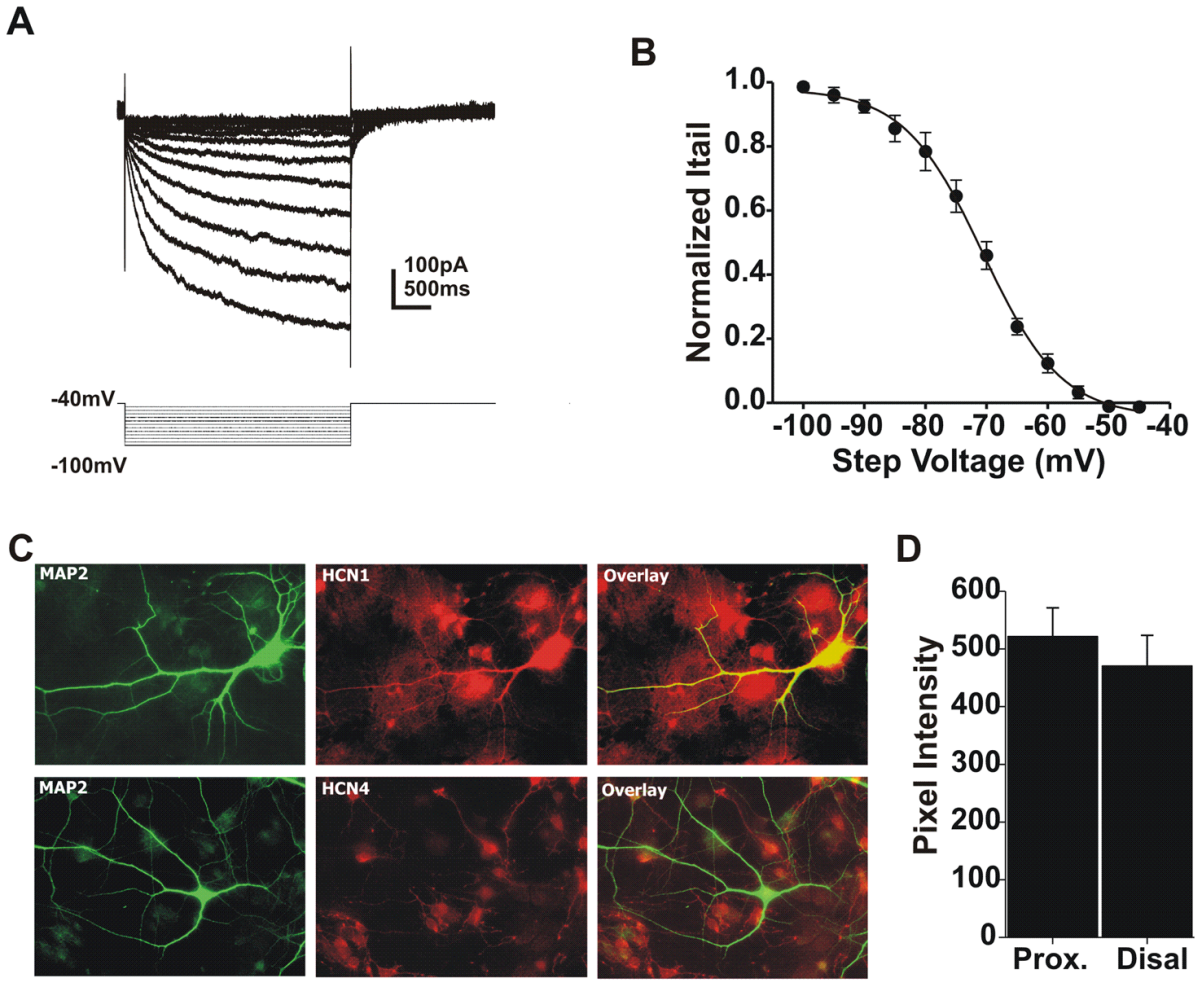
Retinal ganglion cells express HCN1 and HCN 4 channels. **A**) Traces obtained by subtracting responses to voltage steps (−45–100 mV) before and after application of ZD7288. **B**) A plot of normalized tail current amplitude at each voltage. **C**) Images of cultured ganglion cells showing MAP2 (dendritic marker), and HCN1 or HCN4 staining. **D**) Pixel intensity for proximal and distal regions along the dendrites (paired t-test; p>0.05; N = 115 dendrites; 50 cells).

Although we found that summation is uniform over the length of the dendrite under control conditions (Figure 2), we revisited this question to determine whether HCN channels contributed to this uniformity. As described for Figure 2, three uncaging pulses were delivered to a single location with varying interstimulus intervals (1–125 ms). For proximal dendritic locations (<50 µm) the range of intervals over which supralinear summation occurred was increased from <50 ms to >75 ms as might be expected when summation is increased (Figure 8C; N = 8 cells). However, this effect was much larger in the distal dendrites where all interstimulus intervals produced supralinear summation (Figure 8C). To estimate the amount of HCN active at rest we applied voltage steps from −40 to −100 mV in the absence or presence of ZD7288. IV curves were then created by subtracting the ZD7288 traces from controls and measuring tail current amplitude (Figure 9A, B; N = 11 cells). We found that about 25% of HCN channels are open at −65 mV. These results suggest that HCN acts to compensate for dendritic filtering, imposing the uniformity in summation seen under control conditions.

### Retinal ganglion cells express HCN1 and HCN4

To determine which HCN channel subtype is expressed in the retina, we used fluorescent antibodies to label isoform 1, 2 and 4. We did not test for the presence HCN3 because it is thought to be restricted to cone pedicles [33]. To isolate signals from ganglion cells we immunolabeled HCN channels on cultured ganglion cells. Fluorescently labeled HCN1 channel protein was readily detected in the soma and dendrites of retinal ganglion cells. When overlaid with a dendritic marker antibody, microtubule-associated protein 2 (MAP-2), HCN1 immunoreactivity could be seen throughout the dendritic tree (Figure 9C). Co-labeling for GluR2 subunits allowed us to identify ganglion cells [34], [35]. HCN4 immunoreactivity was also present in the soma and dendrites (Figure 9C). These results agree with earlier findings that both channel subtypes are found in retina [36]. We did not detect immunoreactivity for HCN2 subtype, consistent with previous reports [33], [36], [37]. To quantify expression levels of HCN1 for proximal and distal dendrites we measured pixel intensity in two bins, <50 µm and >100 µm from the soma. We found no clear difference in expression levels for these regions of dendrite (Figure 9D; paired t-test; p>0.05; N = 115 dendrites; 50 cells). However, this type of assay is not highly sensitive and to reliably detect a difference in proximal and distal staining may require a substantial differential, perhaps as high as 50%.

### HCN confers directional summation

**Figure 10:**
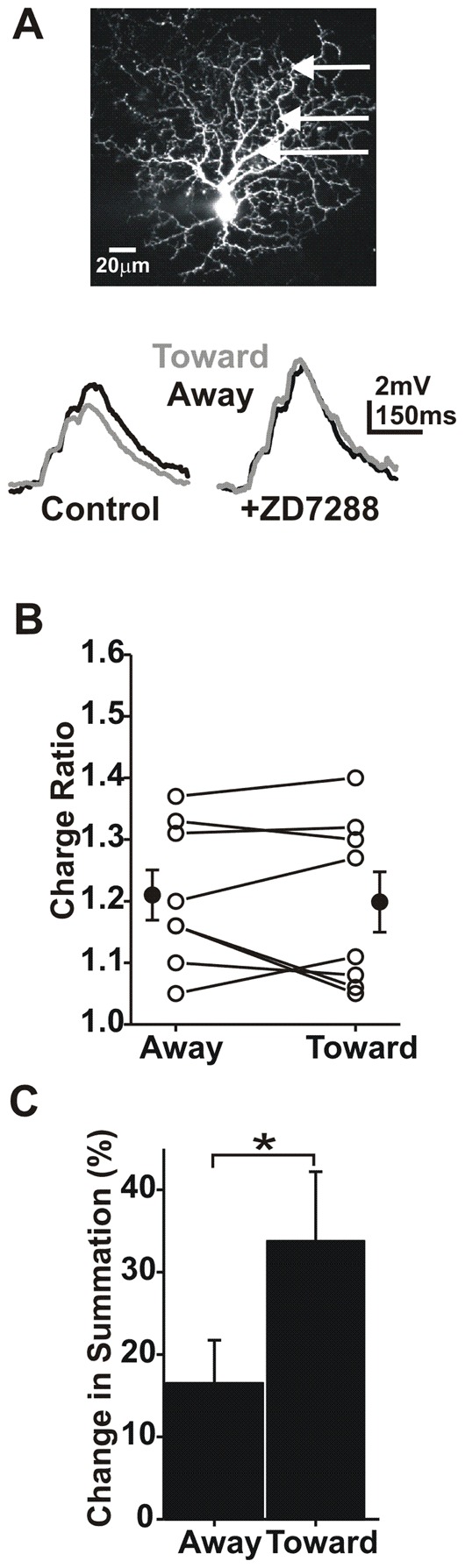
HCN channels confer centrifugal directionality to summation. **A**) Summed EPSPs (average of 8 trials) before and after ZD7288 application. **B**) Plots of the charge ratio (summed depolarization/sum of individual depolarization charges) after application of ZD7288. There was no significant difference in the charge ratio after blockade of HCN (paired t-test, p>0.05; N = 8 cells). **C**) A plot of the change in the charge ratio after channel block.

Blockade of HCN increased summed EPSP charge in both directions (Figure 10A). However, this effect was larger for centripetal than for centrifugal summation, effectively eliminating the directional component of summation. Summation moving toward the soma was increased by 33.8±8.5%, while summation moving away from the soma was only increased by 16.5±5.2% (Figure 10C; paired t-test; p<0.05; N = 8). Under control conditions, the charge ratio was larger for input moving away from the soma (1.24±0.08 away vs. 1.03±0.07 toward; paired t-test, p<0.01; N = 8). After application of ZD7288 the charge ratios were 1.21±0.04 (away) vs. 1.19±0.0.05 (toward; paired t-test; p>0.05; N = 8; Figure 10B). ZD7288 application hyperpolarized resting membrane potential by an average change of −2.4±1 mV which was not significant (paired t-test; p>0.05; N = 8; range 6.5–1 mV). However, it should be noted that small potential changes may be difficult to detect under current clamp conditions due to fluctuation in resting potential. As a result, we conclude that HCN plays an important role in establishing DS.

### Biophysical model of centrifugal Direction Selectivity

#### Simple model

**Figure 11:**
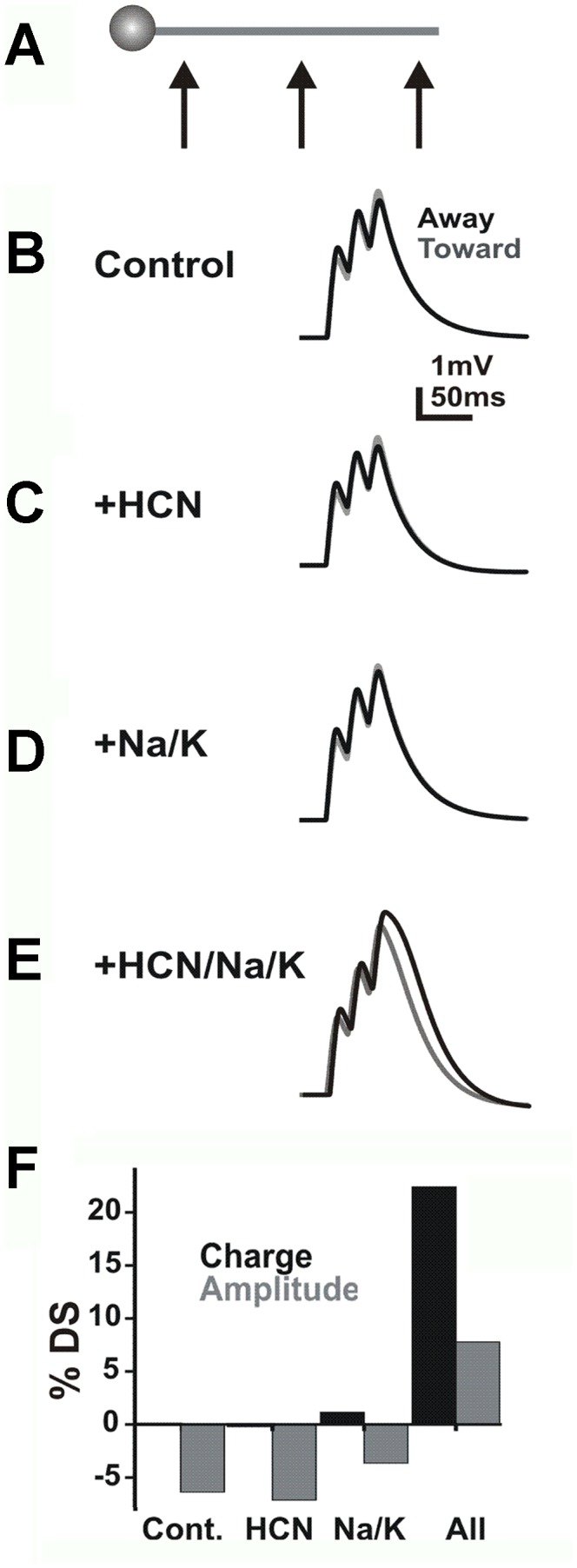
Directional summation in a simple model system. **A**) A cell with an unbranched dendrite was activated at multiple locations to elicit summed EPSPs. **B**) EPSPs for a model without dendritic voltage-dependent channels as measured at the soma. **C**) EPSPs after dendritic HCN channels have been added. **D**) EPSPs after Na/K channels were added to the model. **E**) EPSPS from a model with Na/K/HCN channels. **F**) A plot of the % direction selectivity (DS; [away-toward]/toward*100) for amplitude and charge in each condition from B–E.

To further explore the biophysical properties of HCN channels and summation we created a model which included a cell with an unbranched dendrite and Markov state models of HCN, Na, and K channels in which the voltage offset and the speed of the kinetic rate transition functions could be varied. In addition, the channel densities were specified by location (soma, proximal dendrite, medial dendrite, and distal dendrite). This allowed for the differential activation of individual channel subtypes in specific cell regions (Table 1). The model contained excitatory synapses that originated in presynaptic compartments activated by voltage clamp with the appropriate timing. The synapses were activated in sequence to elicit summed EPSPs from multiple locations along the dendrite (Figure 11A). Somatic EPSPs were analyzed by calculating % DS ([away-toward]/toward*100) for amplitude and charge.

When no voltage-gated channels were included in the computational model, we observed modest DS in amplitude when moving toward the soma (Figure 11B) as previously shown in passive models [22], [38]. In our calibration runs, the addition of HCN channels depolarized Vrest. We typically constrained the depolarization from HCN channels to less than 5 mV to match the real data. The effect of this depolarization was to reduce the summed amplitude and driving force for EPSPs (typically 6% or ∼0.3 mV; Figure 11C). We then tuned the activation of HCN to the real data by changing offset voltages for its kinetic functions to reduce its activation at the peak of ∼5 mV EPSPs while maintaining activation at Vrest. This in turn reduced the amplitude of the EPSPs by a larger fraction than it modified the driving force for Vrest, typically ∼1 mV (Figure 11C). Charge was the same moving away and toward the soma. In the absence of HCN channels, addition of Na and K channels to the model (at low density, 20–30 mS/cm^2^) amplified EPSPs (∼6%) but had little effect on charge or DS (Figure 11D). Because the distal dendrites were partially isolated electrotonically from the soma [3], [39], [40], distal EPSPs (measured at the dendritic activation site) were preferentially amplified.

**Figure 12:**
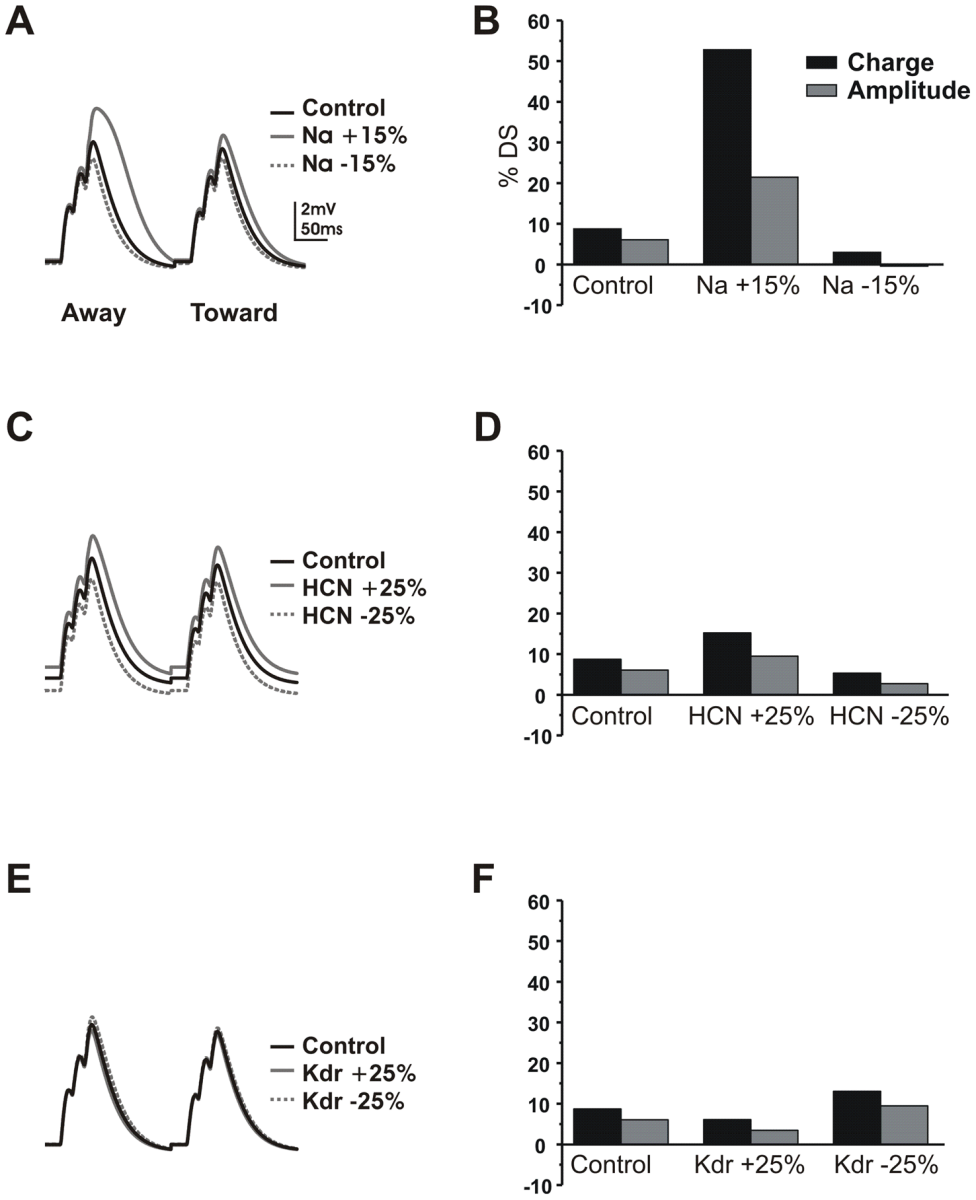
Variations in the parameter set show that DS is robust. A) Varying Na conductance shows its effect in amplifying the directional difference. Left, a 15% increase in Na conductance (solid gray trace) amplified both peaks but more so for the centrifugal direction. A 15% decrease (dashed gray trace) reduced the amplification more for the centrifugal direction. **B)** Summary of charge (black bars) and amplitude (gray bars) differences plotted against % DS. The increase in Na conductance magnified both charge and amplitude differences. The decrease in Na conductance diminished both. **C)** For a 25% change in HCN conductance the DS slightly increased or decreased, due to the shift in resting membrane potential. **D)** Summary of charge and amplitude in response to changes in HCN conductance. **E)** A 25% change in K conductance had the inverse effect of a change in Na conductance, because a larger K conductance canceled the regenerative effects of the Na channels. **F)** Charge and amplitude summary for changes in K conductance.

We also compared a uniform HCN conductance with a gradient having a higher HCN conductance in the distal dendrite and lower in the proximal dendrite and soma. The main result was that the gradient allowed for a higher distal HCN density and increased distal depolarization while maintaining the same Vrest. When Na channels were included in the model, this allowed for further enhancement of local centrifugal DS in the dendritic tip and (to a lesser degree) at the soma. Without the gradient, high proximal HCN produced a substantial change in Vrest (Figure 12C). As a result, we used a gradient of HCN channel conductance, consistent with results from the ganglion cells where activation of HCN produced no significant change in Vrest (Figure 8A).

Inclusion of Na and K channels in addition to HCN channels to the model produced a robust centrifugal DS in both EPSP charge and amplitude similar to that seen experimentally (Figure 11E, F). The Na channels depolarized the distal dendrites by several mV above the depolarization from HCN. However, we tuned the Na and K densities to avoid spike initiation. The K channel density was adjusted in proportion with the Na density to give a Vrest of approximately −64 mV. Thus, the DS we observed in the model was also dependent on subthreshold activation of Na channels in the distal dendrites. DS was robust for Na conductance densities in the range of 15–35 mS/cm^2^. Similarly, DS was robust for 25% changes in HCN and K channel density (Figure 12). These results suggest that centrifugal DS can be created by co-activation of multiple voltage-dependent channels.

#### Ganglion cell model

**Figure 13:**
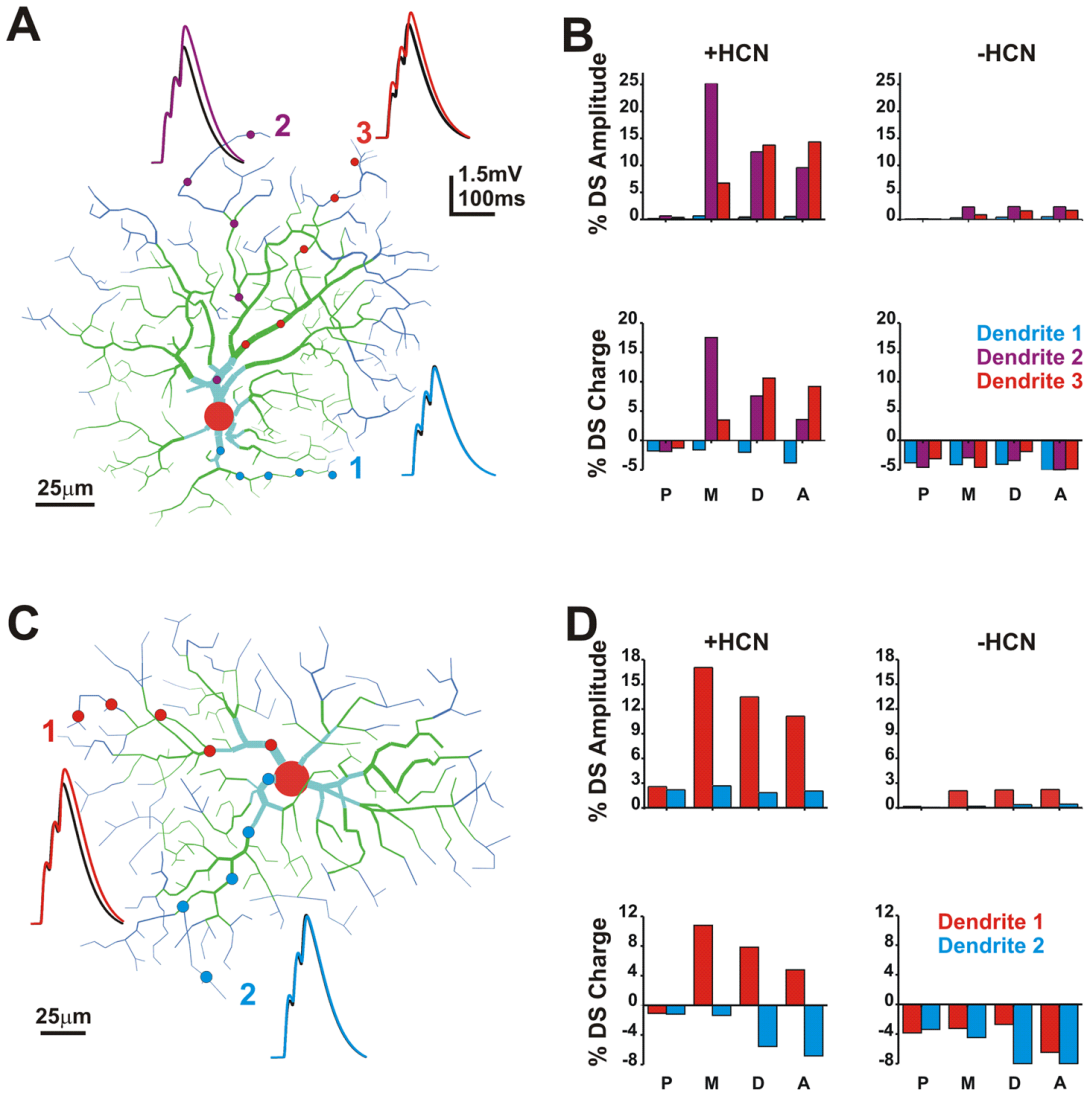
HCN modulates directional summation. **A**) A model of the cell in Figure 10. Circles indicate input sites. Traces are for activation of the middle (M) 3 locations moving away from the soma (color) or toward the soma (black). **B**) Plots of directional summation (DS) ([away-toward]/toward*100) for amplitude and charge. P = 3 most proximal locations, M = middle 3 locations, D = 3 most distal locations, A = alternating locations (distal, middle, proximal). **C**) A model of another cell and activation of two dendrites. **D**) Plots of DS for both dendrites.

We also constructed a more complex model that included Na/K/HCN channels using ganglion cell dendritic morphology derived from the live images collected in our electrophysiology experiments. We stimulated the model with excitatory synapses at five input locations to determine how synapse location affected summation properties (Figure 13A, C). We tested four activation configurations: 1) the three most proximal locations, 2) the middle three locations, 3) the three most distal, and 4) alternating locations (locations 1, 3, 5). Multiple dendrites within the same cell were modeled and analyzed for DS.

As in Figure 3 we observed centrifugal DS in multiple dendrites in the model when HCN channels were present. Exceptions to this result can be found in Figure 13A where dendrite 1 shows no DS for any of the location sequences tested. What distinguished this dendrite from the others is that all activation sites are located close to the soma. In fact, proximal (<100 µm from soma) activation sequences failed to elicit DS for all cells and dendrites tested in the model. These results are consistent with higher HCN current density in the distal dendrites and a role for HCN in DS. Longer dendrites (dendrites 2 and 3) with more distal activation sites (middle, distal, alternating) show centrifugal DS for EPSP charge and amplitude (Figure 13A, B). DS was dependent on the diameter of the dendrites. Occasionally, we observed long dendrites which had limited DS or even minor centripetal DS (Figure 13C, D dendrite 2). This was due to smaller dendritic diameter (<0.3 µm), which resulted in greater isolation from the somatic compartment. Although the thin dendritic tips had a higher input resistance and generated greater DS locally, this DS did not transfer to the soma because of the high axial resistance. Similar results were obtained by reducing the axial resistivity (Ri). In this case, the dendrite was not sufficiently isolated from the soma to allow for generation of the local compartment necessary for generation of DS [22]. Overall, this reduced DS in very fine dendrites from the simulations showed that the principle is a general one [22], [38].

When HCN channels were removed from the model to simulate application of ZD7288, centrifugal DS was eliminated or reversed (Figure 13 B, D). This was true for both EPSP amplitude and charge measures. Dendrites showing no DS with HCN channels were unaffected by removal of HCN. These results further support a role for HCN channels, in combination with voltage-dependent Na and K channels, in establishing a radial/centrifugal DS in traditionally non-direction selective ganglion cells.

## Discussion

### Directional summation

The unique integration properties of On ganglion cells differentiate them from cortical pyramidal and hippocampal neurons ([14] and Figure 4). Glutamate responses recorded at the soma are summed in a supralinear manner for centrifugal input arriving at frequencies greater than 20 Hz (1–50 ms delays). Lower frequency input (75–150 ms delays) sums in a linear fashion. This DS translates to a 3-fold increase in spike probability for centrifugal input for the subthreshold stimuli used in our experiments, suggesting that the direction-dependent increase in summation has significant impact on cell excitability and function in retinal ganglion cells. Centrifugal DS could also facilitate back propagation of action potentials despite the relatively branched configuration of ganglion cell dendritic trees [41]. Active dendritic back propagation of action potentials is necessary in large ganglion cells to allow them to fire at low rates [7], [8].

Here we examined dendritic integration in On ganglion cells. These cells were chosen based on large soma size and our sample is likely to contain multiple On cell subtypes. ∼85% of the cells tested showed centrifugal DS, suggesting that DS is a property of multiple On cell subtypes. In fact, we observed similar DS for Off (N = 4) and On/Off cells (N = 6). These cells were also dependent on HCN for expression of DS. While our sample is insufficient to assess more subtle differences such as the magnitude of DS, we find that a broad range of ganglion cell subtypes use similar dendritic integration strategies. In our culture experiments it was not possible to determine cell subtype and we assume this sample also contains multiple subtypes. As a result, we find that DS is a general property of ganglion cells, used by a broad range of cell subtypes.

### NMDA receptors and summation

Previous work in cortical neurons suggests that the directional component of summation was due to activation of NMDA receptors [14]. However, we found that while NMDA receptor blockade reduced summation, it had no effect on the centrifugal DS in ganglion cells (Figure 7B). Although virtually all classes of ganglion cells express NMDA receptors, differences in subunit composition may preferentially increase summation in specific ganglion cell types. For example, NMDARs that contain the NR2B subunit decay more slowly than NR2A-containing NMDARs [42], and there is evidence that ON ganglion cells preferentially express NR2B-containing NMDARs [43], [44]. Accordingly, one might expect to find that blocking NMDARs will reduce temporal summation to a greater degree in On cells than Off cells. It will be particularly interesting to determine if temporal summation in the dendrites of On/Off ganglion cells ramifying in sublamina b is further reduced by NMDA block compared with dendrites in sublamina a. Differences in temporal summation in the On and Off pathway may contribute to asymmetries in the On and Off pathways [45].

### HCN current density

Dendritic HCN channels lower input resistance, speed the decay of post synaptic potentials, and reduce EPSP summation. The higher HCN conductance we observe in the distal dendrites allows the cell to compensate for EPSP broadening due to dendritic filtering. Consistent with this observation, we find that EPSP responses are surprisingly uniform along and between dendrites of retinal ganglion cells (Figure 2), independent of input location [26]. Such a mechanism insures that input strength is accurately represented at the soma. As a result, EPSPs have similar kinetics independent of input location along the dendrite.

The mechanism underlying higher HCN conductance in distal dendrites is unclear. One possibility is that there are more channels expressed on distal dendrites. While our immunohistochemistry quantification did not show such an expression gradient, this assay may not be sensitive enough to detect small differences in channel expression. However, it is also possible that channel expression is uniform but channel properties are differentially regulated by the availability of accessory subunits, non-uniform expression of additional channels, or regulation by ligand-gated channels. For example, TRIP8b interacts with the carboxyl-terminal region of HCN channels and regulates their cell-surface expression level and cyclic nucleotide dependence [46]. KV7 or KCNQ channels and CaV3 (T-type) Ca2+ channels also indirectly affect the activation of HCN channels through modulation of membrane potential and resistance [47]–[49]. The neurotransmitter dopamine has also been shown to modulate HCN channels via D1 receptors and cAMP [50]. This second mechanism is consistent with our modeling data which demonstrates that HCN by itself is not sufficient to create the centrifugal DS, additional channels are required. This may also explain why cortical pyramidal neurons, which also express HCN channels in a gradient [51], demonstrate centripetal rather than centrifugal DS [31]. Perhaps these neurons do not express the appropriate channel partners found in ganglion cells.

### HCN and directional summation

We find that HCN channel conductance is higher in distal dendrites (Figure 7) and that HCN channels are necessary for expression of DS as blockade eliminates DS (Figure 10). Under control conditions the depolarization caused by a synaptic input spreads passively along the dendrites, closing HCN channels. While this is true for both the distal and proximal dendrites, proximal dendrites have a low HCN conductance. As a result, the spreading depolarization does very little to amplify the proximal EPSP. In the distal dendrites, HCN is high and there is a substantial increase in EPSP charge. A further source of amplification in the distal dendrites is suggested by our model which shows that Na and K channels are also required for DS. Larger distal EPSPs can in turn activate Na channels which could further increase distal EPSP amplification.

In addition, the relatively small diameter of distal dendrites results in greater electrotonic isolation, increased input resistance, larger local EPSPs and increased activation of Na channels. We found that this effect is further enhanced when HCN channels are concentrated in the distal dendrites, preferentially depolarizing them. But the greater electrotonic isolation of very fine distal dendrites prevents transfer of their local DS to the soma. Thus, the model suggests that the DS effect measured at the soma depends on partial electrotonic isolation of distal dendrites [22].

However, this model depends on EPSPs of sufficient size to activate the channels and we used relatively small subthreshold EPSPs. To further assess the role of voltage gated Na channels in DS, we repeated our experiments in the presence of a low TTX concentration (0.1 µM). We used a low dose of TTX to avoid global blockade of all network Na channels which could result in complex outcomes for the cell that would be difficult to tease apart. TTX reduced single EPSP amplitude, confirming activation of Na channels under our baseline conditions. However, paradoxically, it had no effect on DS which remained intact (N = 6 cells; data not shown). While these results might suggest voltage gated Na channels are not involved in DS, the experiment could be compromised by the presence of unblocked Na channels (due to the low dose of TTX), and TTX-insensitive Na channels which have been demonstrated in retina. Further, we have only examined a limited number of channel types and there may be additional channels involved. Overall, we have demonstrated that DS can be achieved by a combination of voltage-dependent channels, independent of asymmetric presynaptic innervation.

### A general strategy for summation in the retina

Over the course of evolution retinal ganglion cells acquired unique properties to perform complex functional roles such as sensing movement and direction of motion through natural environments. Perhaps the first step toward detecting motion was to bias retinal ganglion cells toward enhancing EPSP summation for responses to centrifugal motion. Mechanistically, this function can be accomplished by non-uniform dendritic distribution or activation of ion channels. Our simulations suggest that the centrifugal bias is a consequence of partial isolation of the ganglion cell's distal dendrites that contain voltage-gated Na, K, and HCN channels [22], [26]. The Na channels, necessary to support active back-propagation of spikes into the distal dendrites for control of spike frequency [7], [8], also amplify subthreshold PSPs in the distal dendrites with the aid of depolarization from HCN channels. Thus, the voltage-gated channels also tend to amplify the larger distal PSPs evoked by centrifugal motion. This raises the question of whether such mechanisms, which might equalize responses to light across the ganglion cell's receptive field, or generate DS which might preferentially code looming objects, could be important for vision.

Our results cannot answer these questions, but they highlight possible evolutionary routes for the development of direction selectivity. As more complex retinal networks formed later in time more specific features of movement were coded by layering additional mechanisms. For example, locust retina contains specialized ganglion cells that detect looming stimuli [52]. Voltage-dependent channels involved in spike accommodation are thought to underlie this ability [52]. One prediction of this model is that On/Off cells, which are better suited for detection of looming stimuli, will have a higher degree of supralinear summation than other ganglion cell types. The addition of asymmetrical inhibition [53] to these cells could then allow for detection of directional motion. Judicious combination of directional tuning mechanisms within a given cell could allow for a broad array of detectors assembled from basic ganglion cell building blocks.

## Supporting Information

Figure S1 **Directional summation in ganglion cell dendrites.** **A**) A plot of the peak amplitudes for summed depolarizations moving away from or toward the soma (4.85±0.63 mV away; 4.25±0.56 mV toward) **B**) A plot of the total charge for summed depolarizations (782.1±79.1 pA/ms away; 637.2±75.8 pA/ms toward). *paired t-test; p<0.05; N = 13 cells.
(TIF)
