# Supplementary figures and images for: Directional Summation in Non-direction Selective Retinal Ganglion Cells
Source: PLoS Comput Biol. 2013 Mar 14;9(3):e1002969. doi: 10.1371/journal.pcbi.1002969 (PMC3597528; doi:10.1371/journal.pcbi.1002969)

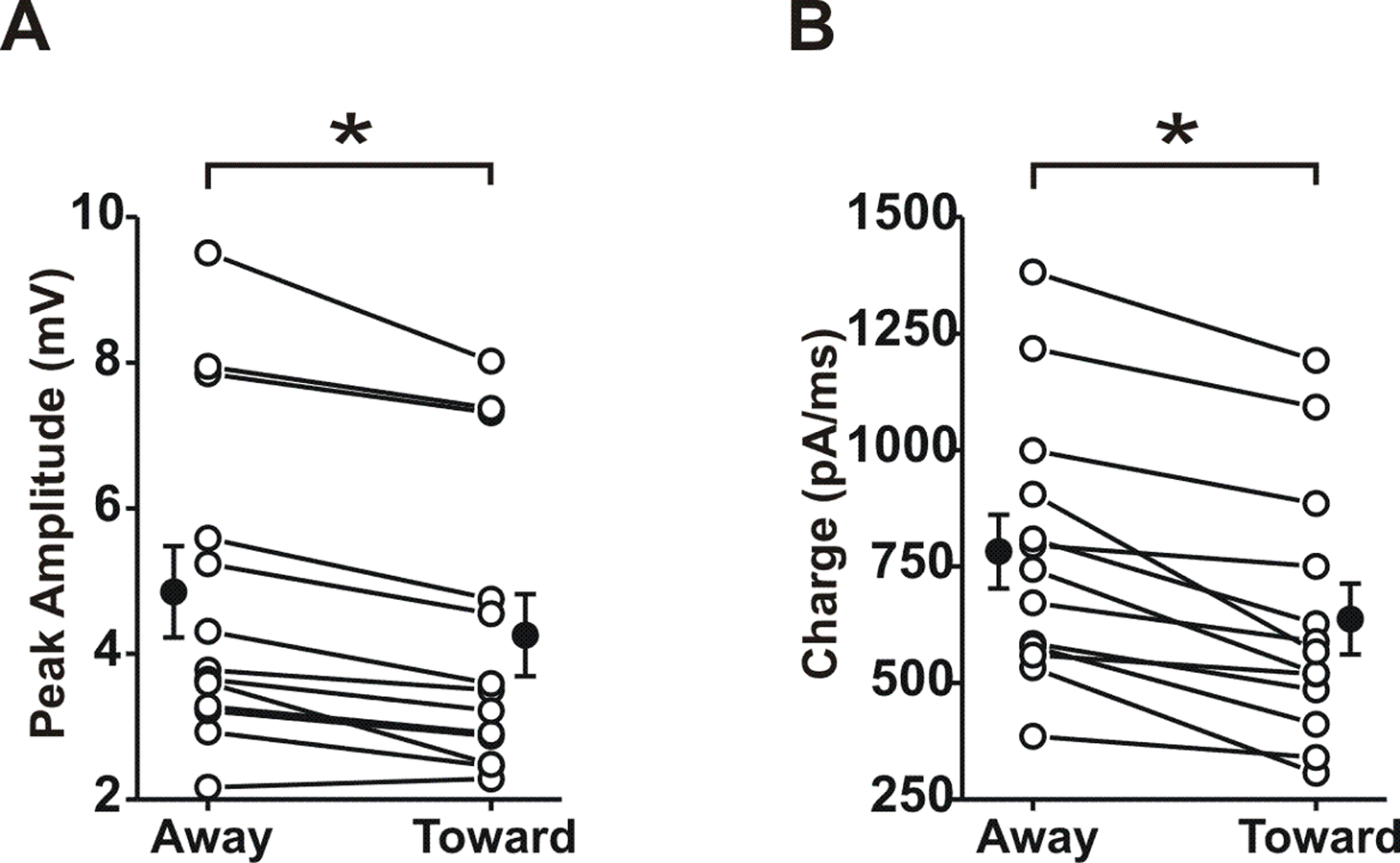

Supplement: Figure S1 — Directional summation in ganglion cell dendrites. A) A plot of the peak amplitudes for summed depolarizations moving away from or toward the soma (4.85±0.63 mV away; 4.25±0.56 mV toward) B) A plot of the total charge for summed depolarizations (782.1±79.1 pA/ms away; 637.2±75.8 pA/ms toward). *paired t-test; p<0.05; N = 13 cells. (TIF) [file pcbi.1002969.s001.tif]
